# Supplementary material for: Identification of Accurate Reference Genes for qRT-PCR Analysis of Gene Expression in Eremochloa ophiuroides under Multiple Stresses of Phosphorus Deficiency and/or Aluminum Toxicity
Source: Plants (Basel). 2023 Nov 2;12(21):3751. doi: 10.3390/plants12213751 (PMC10649868; doi:10.3390/plants12213751)
Supplement: Supplementary file 1 [file plants-12-03751-s001.zip › FigureS2.pdf]

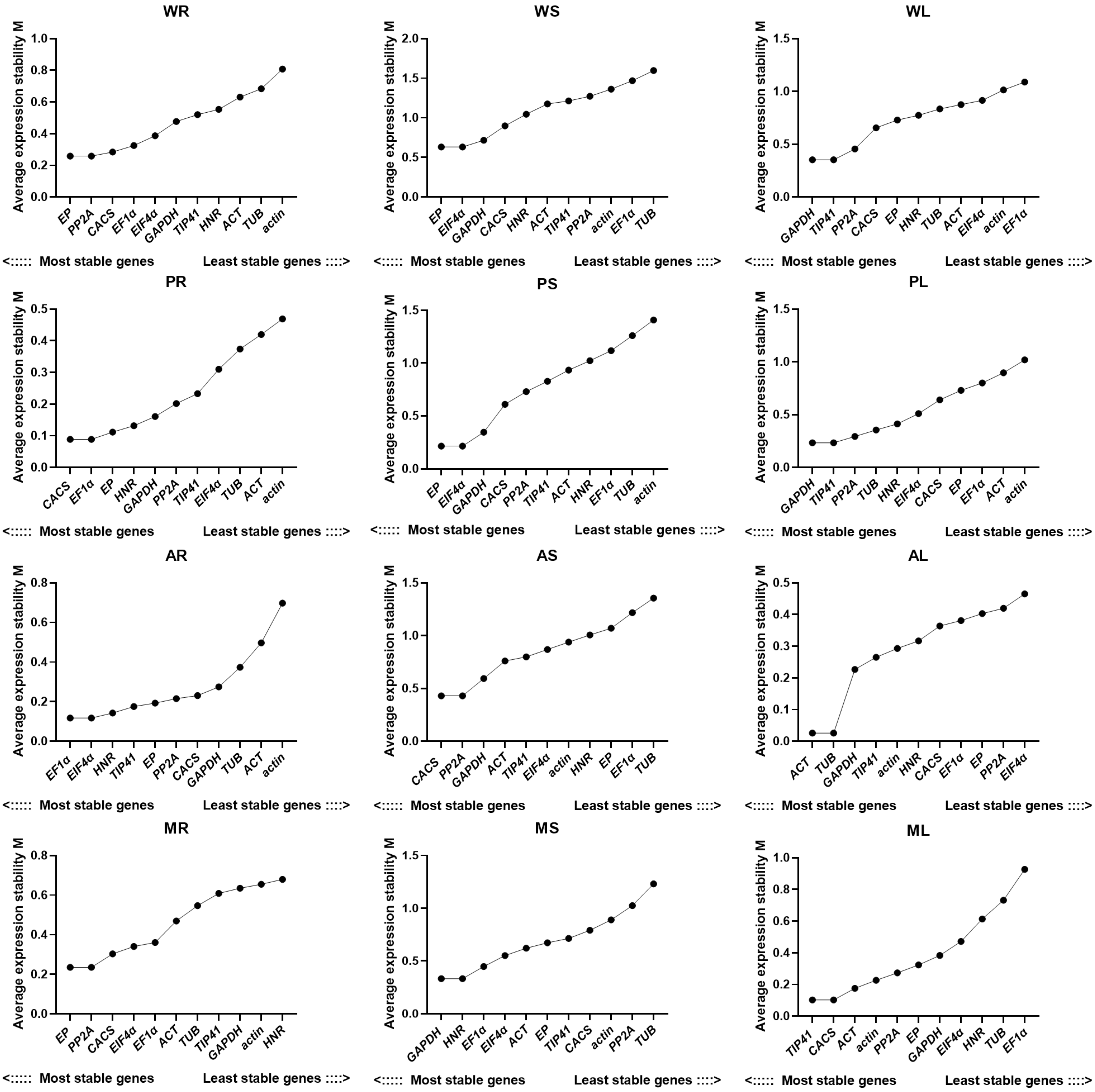
WR
Average expression stability M
EP PP2A CACS EF1α EIF4α GAPDH TIP41 HNR ACT TUB actin
<::::: Most stable genes
Least stable genes ::::>
WS
Average expression stability M
EP EIF4α GAPDH CACS HNR ACT TIP41 PP2A actin EF1α TUB
<::::: Most stable genes
Least stable genes ::::>
WL
Average expression stability M
GAPDH TIP41 PP2A CACS EP HNR TUB ACT EIF4α actin EF1α
<::::: Most stable genes
Least stable genes ::::>
PR
Average expression stability M
CACS EF1α EP HNR GAPDH PP2A TIP41 EIF4α TUB ACT actin
<::::: Most stable genes
Least stable genes ::::>
PS
Average expression stability M
EP EIF4α GAPDH CACS PP2A TIP41 ACT HNR EF1α TUB actin
<::::: Most stable genes
Least stable genes ::::>
PL
Average expression stability M
GAPDH TIP41 PP2A TUB HNR EIF4α CACS EP EF1α ACT actin
<::::: Most stable genes
Least stable genes ::::>
AR
Average expression stability M
EF1α EIF4α HNR TIP41 EP PP2A CACS GAPDH TUB ACT actin
<::::: Most stable genes
Least stable genes ::::>
AS
Average expression stability M
CACS PP2A GAPDH ACT TIP41 EIF4α actin HNR EP EF1α TUB
<::::: Most stable genes
Least stable genes ::::>
AL
Average expression stability M
ACT TUB GAPDH TIP41 actin HNR CACS EF1α EP PP2A EIF4α
<::::: Most stable genes
Least stable genes ::::>
MR
Average expression stability M
EP PP2A CACS EIF4α EF1α ACT TUB TIP41 GAPDH actin HNR
<::::: Most stable genes
Least stable genes ::::>
MS
Average expression stability M
GAPDH HNR EF1α EIF4α ACT EP TIP41 CACS actin PP2A TUB
<::::: Most stable genes
Least stable genes ::::>
ML
Average expression stability M
TIP41 CACS ACT actin PP2A EP GAPDH EIF4α HNR TUB EF1α
<::::: Most stable genes
Least stable genes ::::>
