# Supplementary material for: Identification of Accurate Reference Genes for qRT-PCR Analysis of Gene Expression in Eremochloa ophiuroides under Multiple Stresses of Phosphorus Deficiency and/or Aluminum Toxicity
Source: Plants (Basel). 2023 Nov 2;12(21):3751. doi: 10.3390/plants12213751 (PMC10649868; doi:10.3390/plants12213751)
Supplement: Supplementary file 1 [file plants-12-03751-s001.zip › TableS1.pdf]

**Table S1**. Expression stability values in *Eremochloa ophiuroides* under various treatments determined with NormFinder software.

| | Rank | 1 | 2 | 3 | 4 | 5 | 6 | 7 | 8 | 9 | 10 | 11 |
|---|---|---|---|---|---|---|---|---|---|---|---|---|
| **Total** | gene | ***GAPDH*** | ***HNR*** | ***EIF4α*** | ***CACS*** | ***TIP41*** | ***PP2A*** | ***ACT*** | ***EF1α*** | ***EP*** | ***TUB*** | ***actin*** |
| | Stability | 0.382 | 0.652 | 0.777 | 0.823 | 0.871 | 0.912 | 1.314 | 1.354 | 1.387 | 1.479 | 1.627 |
| **WR** | gene | ***CACS*** | ***GAPDH*** | ***EF1α*** | ***PP2A*** | ***EIF4α*** | ***EP*** | ***TIP41*** | ***HNR*** | ***TUB*** | ***ACT*** | ***actin*** |
| | Stability | 0.16 | 0.317 | 0.426 | 0.468 | 0.473 | 0.537 | 0.568 | 0.623 | 0.653 | 0.662 | 1.289 |
| **PR** | gene | ***EF1α*** | ***GAPDH*** | ***CACS*** | ***EP*** | ***HNR*** | ***PP2A*** | ***TIP41*** | ***EIF4α*** | ***TUB*** | ***ACT*** | ***actin*** |
| | Stability | 0.076 | 0.122 | 0.145 | 0.188 | 0.216 | 0.269 | 0.402 | 0.429 | 0.471 | 0.561 | 0.635 |
| **AR** | gene | ***CACS*** | ***GAPDH*** | ***PP2A*** | ***EF1α*** | ***TIP41*** | ***EIF4α*** | ***HNR*** | ***TUB*** | ***EP*** | ***ACT*** | ***actin*** |
| | Stability | 0.099 | 0.141 | 0.297 | 0.319 | 0.352 | 0.363 | 0.424 | 0.434 | 0.505 | 0.829 | 1.568 |
| **MR** | gene | ***CACS*** | ***TUB*** | ***EP*** | ***GAPDH*** | ***EF1α*** | ***TIP41*** | ***actin*** | ***ACT*** | ***PP2A*** | ***EIF4α*** | ***HNR*** |
| | Stability | 0.152 | 0.443 | 0.47 | 0.489 | 0.518 | 0.526 | 0.53 | 0.533 | 0.542 | 0.557 | 0.691 |
| **WS** | gene | ***GAPDH*** | ***HNR*** | ***EIF4α*** | ***EP*** | ***ACT*** | ***CACS*** | ***TIP41*** | ***actin*** | ***PP2A*** | ***EF1α*** | ***TUB*** |
| | Stability | 0.365 | 0.69 | 0.801 | 0.93 | 1.01 | 1.033 | 1.171 | 1.404 | 1.446 | 1.462 | 1.973 |
| **PS** | gene | ***TIP41*** | ***ACT*** | ***GAPDH*** | ***HNR*** | ***PP2A*** | ***EIF4α*** | ***EF1α*** | ***CACS*** | ***EP*** | ***TUB*** | ***actin*** |
| | Stability | 0.303 | 0.41 | 0.609 | 0.717 | 0.963 | 0.993 | 1.072 | 1.135 | 1.147 | 1.715 | 1.873 |
| **AS** | gene | ***GAPDH*** | ***EIF4α*** | ***CACS*** | ***HNR*** | ***EP*** | ***PP2A*** | ***actin*** | ***ACT*** | ***TIP41*** | ***EF1α*** | ***TUB*** |
| | Stability | 0.111 | 0.153 | 0.372 | 0.709 | 0.786 | 0.829 | 1.052 | 1.316 | 1.327 | 1.488 | 1.878 |
| **MS** | gene | ***GAPDH*** | ***ACT*** | ***TIP41*** | ***EP*** | ***HNR*** | ***EIF4α*** | ***CACS*** | ***EF1α*** | ***actin*** | ***PP2A*** | ***TUB*** |
| | Stability | 0.166 | 0.254 | 0.413 | 0.497 | 0.583 | 0.722 | 0.758 | 0.802 | 1.115 | 1.618 | 2.073 |
| **WL** | gene | ***PP2A*** | ***HNR*** | ***CACS*** | ***TIP41*** | ***EP*** | ***GAPDH*** | ***TUB*** | ***EIF4α*** | ***ACT*** | ***actin*** | ***EF1α*** |
| | Stability | 0.381 | 0.477 | 0.539 | 0.598 | 0.599 | 0.647 | 0.807 | 0.842 | 1.038 | 1.237 | 1.26 |
| **PL** | gene | ***HNR*** | ***PP2A*** | ***TUB*** | ***GAPDH*** | ***EIF4α*** | ***CACS*** | ***TIP41*** | ***EP*** | ***EF1α*** | ***ACT*** | ***actin*** |
| | Stability | 0.209 | 0.401 | 0.45 | 0.505 | 0.536 | 0.606 | 0.621 | 0.807 | 0.952 | 1.343 | 1.474 |
| **AL** | gene | ***GAPDH*** | ***HNR*** | ***TIP41*** | ***actin*** | ***PP2A*** | ***EF1α*** | ***CACS*** | ***TUB*** | ***ACT*** | ***EP*** | ***EIF4α*** |
| | Stability | 0.098 | 0.146 | 0.236 | 0.258 | 0.335 | 0.365 | 0.37 | 0.382 | 0.4 | 0.451 | 0.608 |
| **ML** | gene | ***actin*** | ***EP*** | ***CACS*** | ***TIP41*** | ***ACT*** | ***PP2A*** | ***HNR*** | ***GAPDH*** | ***TUB*** | ***EIF4α*** | ***EF1α*** |
| | Stability | 0.077 | 0.163 | 0.196 | 0.272 | 0.298 | 0.488 | 0.728 | 0.774 | 0.973 | 1.097 | 1.784 |
