# Supplementary material for: Identification of Accurate Reference Genes for qRT-PCR Analysis of Gene Expression in Eremochloa ophiuroides under Multiple Stresses of Phosphorus Deficiency and/or Aluminum Toxicity
Source: Plants (Basel). 2023 Nov 2;12(21):3751. doi: 10.3390/plants12213751 (PMC10649868; doi:10.3390/plants12213751)
Supplement: Supplementary file 1 [file plants-12-03751-s001.zip › TableS2.pdf]

Table S2. Stabilities of candidate reference genes ranked by BestKeeper.

	Rank	1	2	3	4	5	6	7	8	9	10	11
**Total**	gene	***PP2A***	***TIP41***	***ACT***	***GAPDH***	***HNR***	***CACS***	***EIF4α***	***EP***	***EF1α***	***actin***	***TUB***
	SD	0.66	0.68	0.83	0.89	0.94	0.97	1.03	1.42	1.44	1.45	1.53
**WR**	gene	***CACS***	***PP2A***	***TIP41***	***EP***	***EF1α***	***ACT***	***EIF4α***	***GAPDH***	***HNR***	***TUB***	***actin***
	SD	0.42	0.43	0.50	0.50	0.57	0.63	0.64	0.65	0.78	0.87	1.27
**PR**	gene	***TIP41***	***CACS***	***EP***	***PP2A***	***EF1α***	***HNR***	***GAPDH***	***ACT***	***EIF4α***	***TUB***	***actin***
	SD	0.19	0.35	0.36	0.37	0.38	0.40	0.49	0.54	0.74	0.82	0.86
**AR**	gene	***CACS***	***TUB***	***PP2A***	***EIF4α***	***EF1α***	***GAPDH***	***TIP41***	***HNR***	***EP***	***ACT***	***actin***
	SD	0.32	0.32	0.41	0.41	0.43	0.48	0.49	0.51	0.62	0.64	1.14
**MR**	gene	***ACT***	***TIP41***	***PP2A***	***CACS***	***GAPDH***	***EP***	***HNR***	***EF1α***	***TUB***	***EIF4α***	***actin***
	SD	0.12	0.33	0.34	0.37	0.41	0.44	0.55	0.59	0.60	0.65	0.65
**WS**	gene	***actin***	***CACS***	***PP2A***	***ACT***	***GAPDH***	***TIP41***	***HNR***	***EIF4α***	***EP***	***EF1α***	***TUB***
	SD	0.69	0.71	0.92	0.93	1.03	1.06	1.06	1.18	1.22	1.58	2.16
**PS**	gene	***ACT***	***PP2A***	***actin***	***TIP41***	***CACS***	***HNR***	***GAPDH***	***EF1α***	***EIF4α***	***EP***	***TUB***
	SD	0.55	0.59	0.71	0.75	0.84	0.95	1.09	1.29	1.31	1.48	1.86
**AS**	gene	***PP2A***	***CACS***	***GAPDH***	***ACT***	***actin***	***TIP41***	***EIF4α***	***HNR***	***EP***	***EF1α***	***TUB***
	SD	0.17	0.19	0.58	0.66	0.68	0.69	0.7	0.76	1.01	1.58	1.87
**MS**	gene	***actin***	***CACS***	***TIP41***	***ACT***	***PP2A***	***EP***	***GAPDH***	***EIF4α***	***HNR***	***EF1α***	***TUB***
	SD	0.29	0.64	0.68	0.77	0.96	1.07	1.12	1.16	1.39	1.39	2.16
**WL**	gene	***TIP41***	***PP2A***	***ACT***	***HNR***	***GAPDH***	***TUB***	***EP***	***CACS***	***EIF4α***	***EF1α***	***actin***
	SD	0.47	0.51	0.51	0.59	0.66	0.75	0.91	0.94	1.00	1.38	1.41
**PL**	gene	***TIP41***	***GAPDH***	***PP2A***	***ACT***	***TUB***	***EIF4α***	***HNR***	***EP***	***CACS***	***EF1α***	***actin***
	SD	0.15	0.23	0.35	0.38	0.43	0.55	0.67	1.14	1.19	1.43	1.64
**AL**	gene	***EP***	***CACS***	***EF1α***	***HNR***	***PP2A***	***GAPDH***	***TIP41***	***actin***	***TUB***	***ACT***	***EIF4α***
	SD	0.09	0.16	0.18	0.24	0.33	0.36	0.38	0.51	0.58	0.6	0.65
**ML**	gene	***EP***	***CACS***	***PP2A***	***ACT***	***TIP41***	***actin***	***HNR***	***GAPDH***	***TUB***	***EIF4α***	***EF1α***
	SD	0.26	0.31	0.33	0.39	0.4	0.45	0.51	0.65	0.72	0.86	1.2
