# Supplementary figures and images for: Identification of Accurate Reference Genes for qRT-PCR Analysis of Gene Expression in Eremochloa ophiuroides under Multiple Stresses of Phosphorus Deficiency and/or Aluminum Toxicity
Source: Plants (Basel). 2023 Nov 2;12(21):3751. doi: 10.3390/plants12213751 (PMC10649868; doi:10.3390/plants12213751)

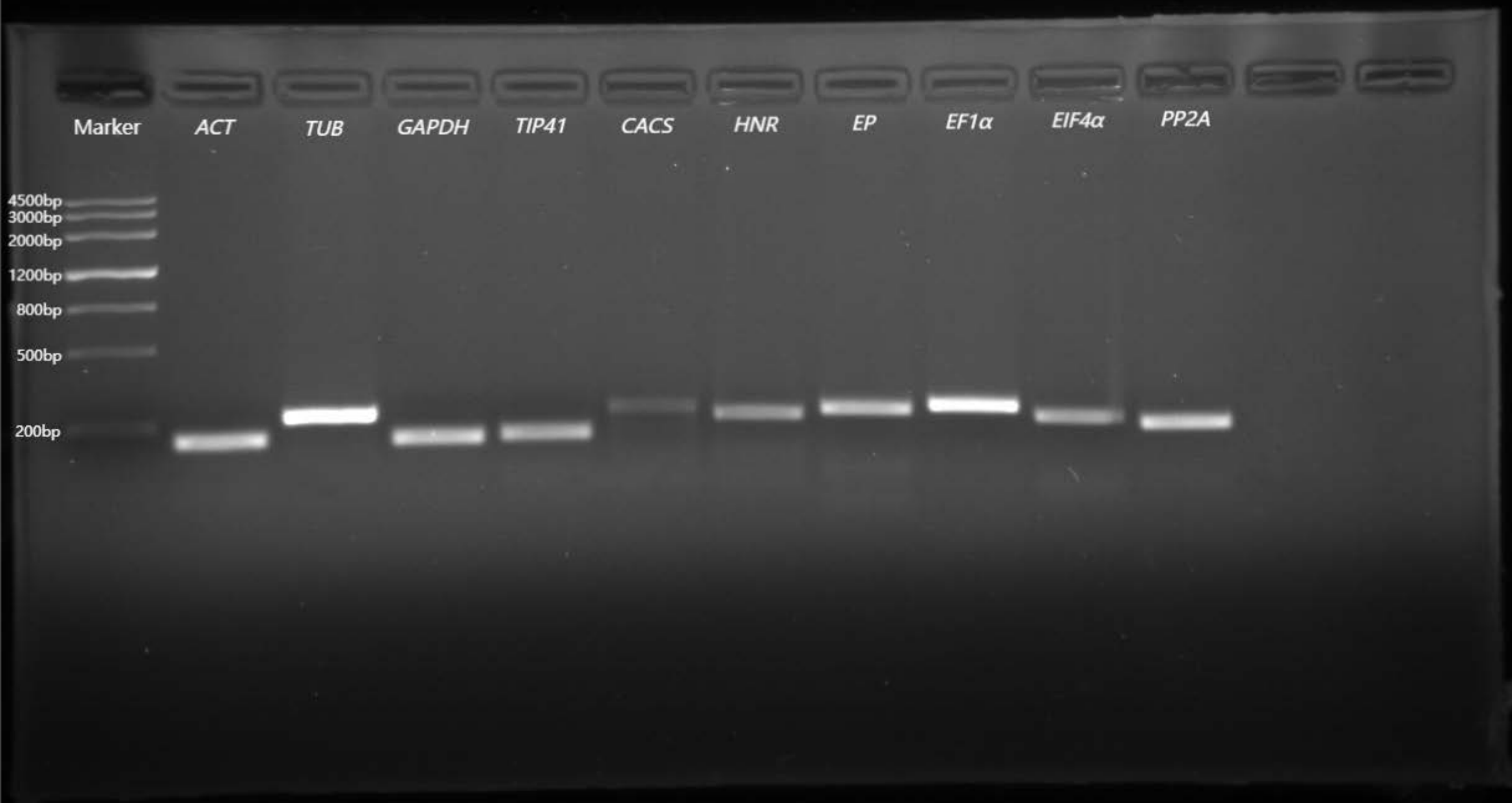

Supplement: Supplementary file 1 [file plants-12-03751-s001.zip › FigureS1.pdf]
